# International survey of De-implementation of initiating parenteral nutrition early in Paediatric intensive care units

**DOI:** 10.1186/s12913-019-4223-x

**Published:** 2019-06-13

**Authors:** Esther van Puffelen, An Jacobs, Charlotte J. M. Verdoorn, Koen F. M. Joosten, Greet van den Berghe, Erwin Ista, Sascha C. A. T. Verbruggen

**Affiliations:** 1 Department of Paediatrics and Paediatric Surgery, Erasmus MC - Sophia Children’s Hospital, University Medical Centre Rotterdam, Wytemaweg 80, 3015 CN Rotterdam, The Netherlands; 2 Department of Cellular and Molecular Medicine, Clinical Division and Laboratory of Intensive Care Medicine, University Hospitals KU Leuven, Leuven, Belgium; 3 Department of Internal Medicine, Nursing Science, Erasmus MC, University Medical Centre Rotterdam, Rotterdam, The Netherlands

**Keywords:** De-implementation, Survey, Questionnaire, Parenteral nutrition, Intensive care units, pediatric, Nutritional support

## Abstract

**Background:**

Initiating parenteral nutrition (PN) within 24 h in critically ill children is inferior to withholding PN during the first week, as was found in the PEPaNIC study. The aims of this study were to investigate de-implementation of early initiation of PN at PICUs worldwide, and to identify factors influencing de-implementation.

**Methods:**

A cross-sectional online survey was conducted (May – October 2017), consisting of 41 questions addressing current PN practices, the degree of de-implementation, and factors affecting de-implementation.

**Results:**

We analysed 81 responses from 39 countries. Of these 81 respondents, 53 (65%) were aware of the findings of the PEPaNIC study, and 43 (53%) have read the article. In these 43 PICUs, PN was completely withheld during the first week in 10 PICUs, of which 5 already withheld PN (12%), and 5 de-implemented early initiation of PN (12%). Partial de-implementation was reported by 17 (40%) and no de-implementation by 16 (37%). Higher de-implementation rates were observed when the interpreted level of evidence and grade of recommendation of PEPaNIC was high. Predominant reasons for retaining early initiation of PN were concerns on withholding amino acids, the safety in undernourished children and neonates, and the long-term consequences. Furthermore, the respondents were waiting for updated guidelines.

**Conclusions:**

One year after the publication of the PEPaNIC trial, only two-thirds of the respondents was aware of the study results. Within this group, early initiation of PN was de-implemented completely in 12% of the PICUs, while 40% asserted partial de-implementation. Increasing the awareness, addressing the intervention-specific questions and more frequently revising international guidelines might help to accelerate de-implementation of ineffective, unproven or harmful healthcare.

**Electronic supplementary material:**

The online version of this article (10.1186/s12913-019-4223-x) contains supplementary material, which is available to authorized users.

## Background

Optimal nutrition is considered essential to improve outcome in the paediatric intensive care unit (PICU) but large well-designed randomised, controlled trials (RCTs) with clinically relevant outcome measures are lacking [1, 2]. The limited evidence leads to a wide variation in nutritional practices between individual intensivists, PICUs and countries. This variation includes timing of and thresholds for the initiation of parenteral nutrition (PN), as measured by a worldwide survey with a point-prevalence [3]. According to this survey completed in 2014, in 20% of the PICUs, PN was initiated within 24 h after admission, and in 55% of the PICUs within 48 h [3]. The international guidelines at that time were based on small studies with surrogate outcome measures, observations, and expert opinion, and could not provide clear recommendations on the timing of initiating PN in critically ill children [4, 5]. In 2016, the results of the large, international, multicentre, RCT ‘PEPaNIC’ (Paediatric Early versus Late Parenteral Nutrition in Intensive Care) were published [6]. This RCT showed that administering PN within 24 h after PICU admission (Early-PN; the standard therapy) was clinically inferior to withholding PN during the first week of PICU admission (Late-PN) [6]. Withholding PN during the first week prevented new infections, shortened intensive care dependency, the duration of mechanical ventilation and hospital stay. Based on the impact of these findings, and the scarcity of evidence for the early use of PN in PICUs, one could expect that currently, initiation of supplemental PN is delayed until after the first week of critical illness in the majority of PICUs.

De-implementation or de-adoption is described as ‘reducing or stopping low-value, ineffective, harmful or unproven care’. [7–9] However, rational and quantitative evidence are only part of the driving forces for decision making and only 49% of the interventions is supported or contradicted by the available evidence [7, 10]. Little is known about the factors that influence the extend and pace of de-implementation [8, 11]. Moreover, currently, only 10% of the de-implementation research has focused on paediatric healthcare [9].

In this study, we explored the degree of early de-implementation of initiating PN in the first week in PICUs and barriers for de-implementation with a survey among physicians and dieticians across PICUs worldwide.

## Methods

This electronic (LimeSurvey GmbH version 2.06lts) cross-sectional survey was conducted between May and October 2017. It consisted of 41 questions and was provided in English, French and Spanish. The full questionnaire used for this survey can be found as online supplement to this article (Additional file 1). In brief, the survey was developed to collect information in different echelons. The first part collected general information of the respondents and responding PICUs, the second part focused on the current practice of PN in the responding PICU, and the third part investigated the awareness of the results of the PEPaNIC trial. Subsequently, the respondents who had read the findings of this study prior to our survey were requested to participate in the final part of the survey in which they were asked to grade the quality of evidence of the PEPaNIC trial according to the Scottish Intercollegiate Guidelines Network (SIGN) system that was provided in the survey [12]. Finally, they were asked whether and how the PEPaNIC results has changed the current practice of initiating PN in their PICU, and which factors have influenced the degree of de-implementation in their PICU.

The survey was piloted by independent clinicians in two different centres (Erasmus MC-Sophia Children’s Hospital, Rotterdam, the Netherlands and the University Hospital of Leuven, Belgium) to test the clarity, relevance and clinical sensibility of the questionnaire, and the questionnaire was adapted accordingly. Data from this pilot were not included in the final analyses and survey results. The survey was electronically distributed among members of the World Federation of Pediatric Intensive and Critical Care Societies (WFPICCS) by newsletter and Twitter, and to specific members of the European Society of Paediatric and Neonatal Intensive Care (ESPNIC). Reminders were sent three times with six-week intervals. If more than one questionnaire was present for a PICU, the answers were weighed by the inverse of the number of completed questionnaires per centre in order to process conflicting statements within one PICU, without disrupting the weight of the answers per PICU.

Main outcome was the degree of de-implementation (fidelity), with complete de-implementation defined as withholding PN until day 8 of PICU admission. Partial de-implementation was defined as postponed initiation of PN (but still initiated prior to day 8 in PICU) and/or decreased amount of PN as compared with nutritional practices before the results from PEPaNIC, or only administering PN during the first week in specific patient groups. Secondary outcomes were supporting factors and barriers for de-implementation.

Statistical analysis was performed using IBM SPSS statistics version 24. All answers were categorical, and were expressed as numbers and proportions.

## Results

### Response

Since the survey was distributed via Twitter, ESPNIC and WFPICCS, with unknown number of PICUs in their databases, the exact number of invited PICUs is unknown. A total of 88 completed questionnaires were received, of which one was removed because the respondent worked in a Neonatal Intensive Care Unit.

**Fig. 1:**
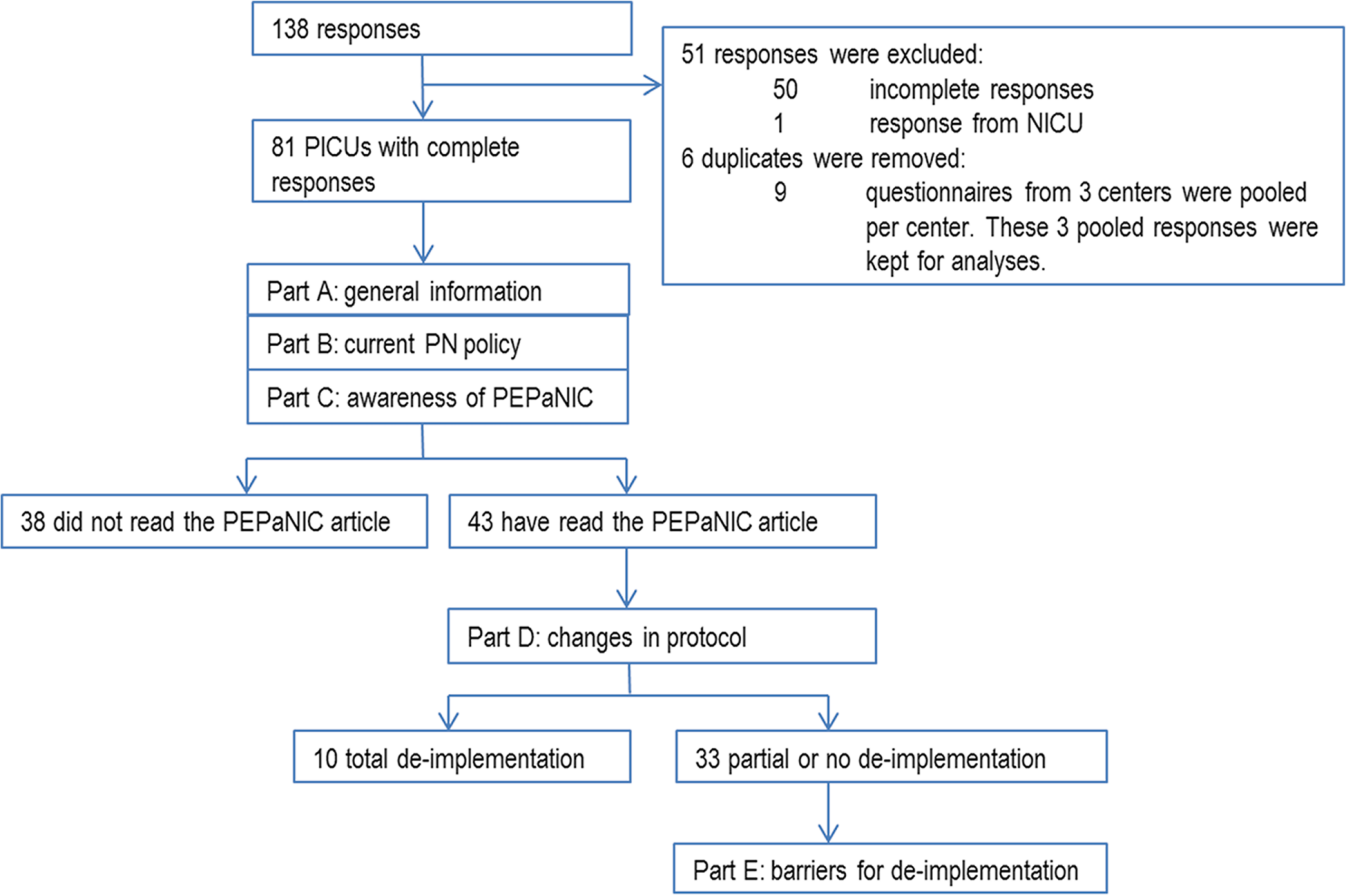
Flowchart of the responses and build-up of the survey. NICU = neonatal intensive care unit; PN = parenteral nutrition

**Fig. 2:**
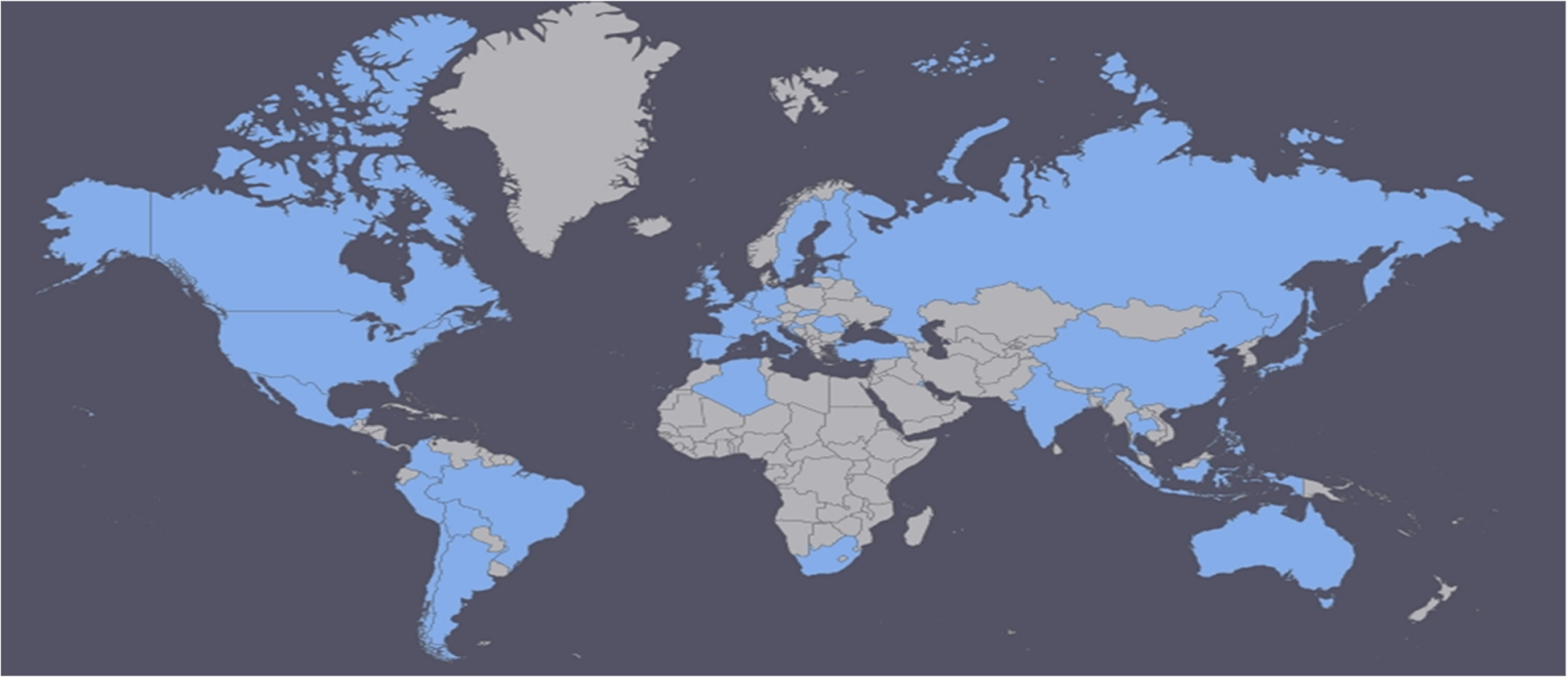
Participating PICUs: 81 responses from 39 countries (in blue), covering six continents. Created with: https://www.amcharts.com/visited_countries/

From the remaining 87 questionnaires, the answers of nine respondents from three centres needed to be pooled per centre by weighing the answers according to the number of completed questionnaires per centre. The 3 pooled responses per centre were kept for analyses, and the individual responses were removed (Fig. 1). Finally, responses from 81 PICUs in 39 countries on 6 continents were analysed (Fig. 2). Of the respondents, 74% were (paediatric) intensivists, 12% were dieticians or nutritionists, 6% were paediatricians, 5% were nurses or nurse practitioners, and 3% were anaesthesiologists.

Of the responding PICUs, 39 (48%) were located in Europe, 14 (17%) in South America, 12 (15%) in North America, and 12 (15%) in Asia (Table 1). The majority of the PICUs had 251–750 paediatric admissions per year (Table 1). All PICU demographics are displayed in Table 1.

**Table 1:** Characteristics of the responding paediatric intensive care units

Characteristic	No. of PICUs (*n* = 81)
Continent	
Europe	39 (48%)
South America	14 (17%)
Asia	12 (15%)
North America	12 (15%)
Africa	2 (3%)
Oceania	2 (3%)
Hospital type	
University children’s hospital	37 (46%)
University hospital	24 (30%)
General hospital	18 (21%)
Other	2 (3%)
Type of PICU	
Multidisciplinary/mixed	75 (93%)
Medical	4 (5%)
Cardiac	1 (1%)
Surgical	1 (1%)
Combination of PICU	
Not combined	66 (82%)
With neonatal ICU	10 (12%)
With adult ICU	4 (5%)
With adult and neonatal ICU	1 (1%)
Size of PICU	
1–10 beds	33 (41%)
11–20 beds	28 (35%)
21–30 beds	16 (50%)
> 30 beds	4 (6%)
Paediatric admissions (patients/year)	
1–250	7 (9%)
251–500	29 (36%)
501–750	18 (22%)
751–1000	7 (9%)
1001–1250	7 (9%)
> 1250	13 (16%)
Mechanically ventilated patients	
< 25%	9 (11%)
25–50%	31 (38%)
50–75%	25 (31%)
> 75%	16 (20%)

*PICU* paediatric intensive care unit, *ICU* intensive care unit

### Current PN practices in PICUs

In 50 of the 81 PICUs (62%), a nutritional protocol regarding PN was present. Most of the protocols were based on international guidelines (27 of 50, 54%), 8 of 50 (16%) on national guidelines, and 15 of 50 (30%) on the opinion of the staff. Respondents from 10 of the 81 PICUs (12%) would always start PN if enteral nutrition (EN) is insufficient, and 4 (5%) would never start PN. In 43 of the 81 PICUs (53%), supplemental PN would be started if enteral nutrition covered less than 80% of the target goals, at 20 (25%) of the PICUs if EN covered less than 50%, and 4 (5%) of the PICUs handled another threshold. PN administration via peripheral intravenous access was possible in 58 of the 81 PICUs (72%).

**Fig. 3:**
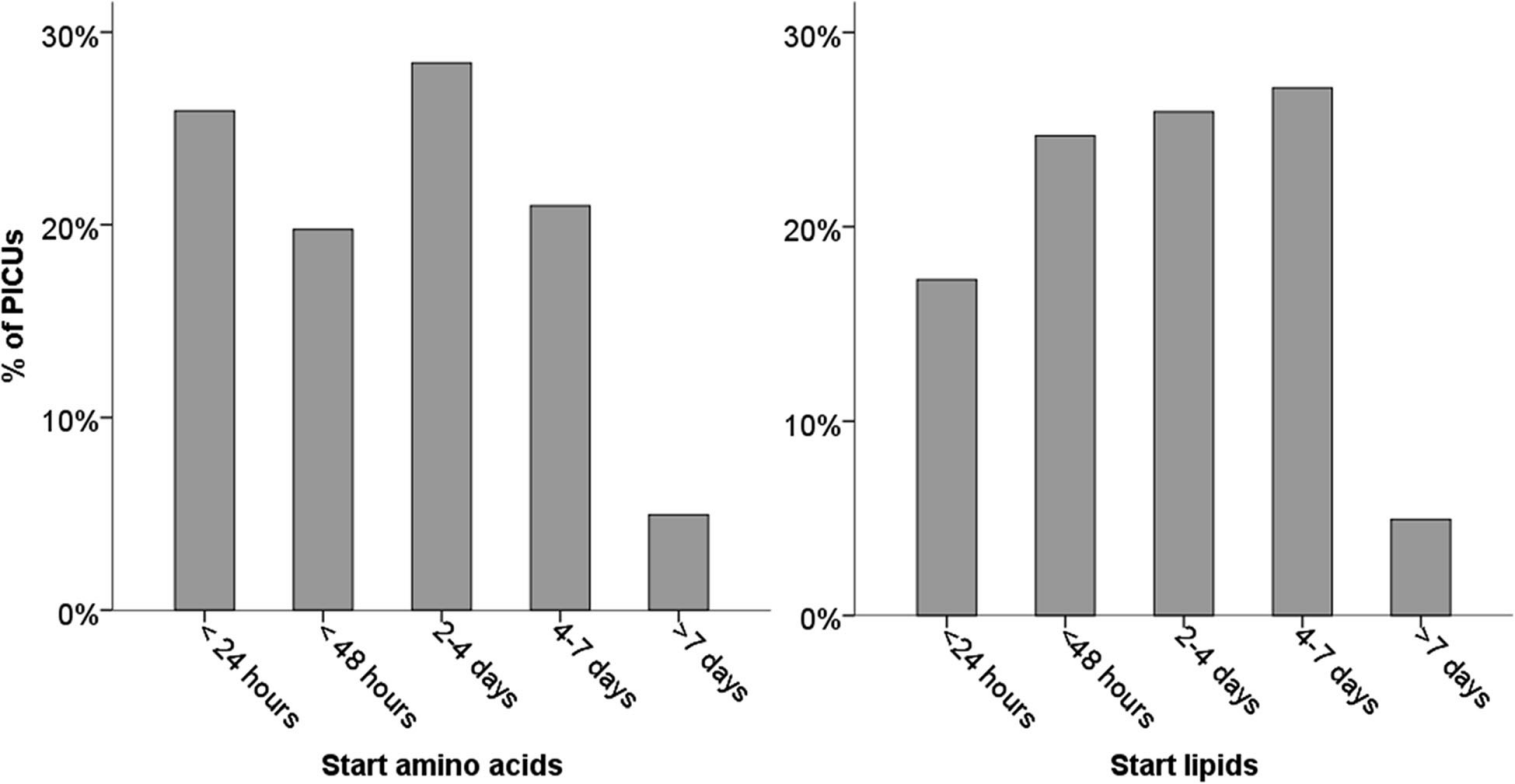
Time to initiate parenteral nutrition when enteral nutrition is (expected to be) insufficient. PICU = paediatric intensive care unit

Regarding the timing of PN initiation, amino acids would be started within 48 h when a child was (expected to be) intolerable to EN in 37 of the 81 PICUs (46%). Initiation of amino acids was postponed beyond the first week in 4 of the 81 PICUs (5%; Fig. 3). Lipids would be started within 48 h in 34 of the 81 PICUs (42%; Fig. 3). Lipids would be initiated beyond the first week in 4 of the 81 PICUs (5%; Fig. 3). Targeted glucose intake during the first 12–24 h varied between 1 and 4 mg/kg/min and 8–10 mg/kg/min. In most cases, 4–6 mg/kg/min was targeted in children who weighed less than 10 kg (38 of 81 PICUs, 47%), 1–4 mg/kg/min in children who weighed 10–30 kg (50 of 81 PICUs, 62%) and also in children weighing more than 30 kg (62 of 81 PICUs, 77%). Of the 81 respondents, 73 (90%) would administer vitamins and trace elements routinely.

### De-implementation of initiating PN early during critical illness

Fifty-three of the 81 respondents (65%) answered to be familiar with the results from the PEPaNIC trial, and 43 (53%) reported to have read the original article. Those who have read the article were larger PICUs and all multidisciplinary/mixed, and reported higher proportions of mechanically ventilated patients (Additional file 2: Table S1). The majority of those who have read the article would start PN if EN was < 50%, whereas the majority of those who have not read the article would start PN if EN was < 80% of target (Additional file 2: Table S1). Furthermore, those who have read the article would start amino acids more often within 48 h than those who did not read the article (Additional file 2: Table S1).

**Fig. 4:**
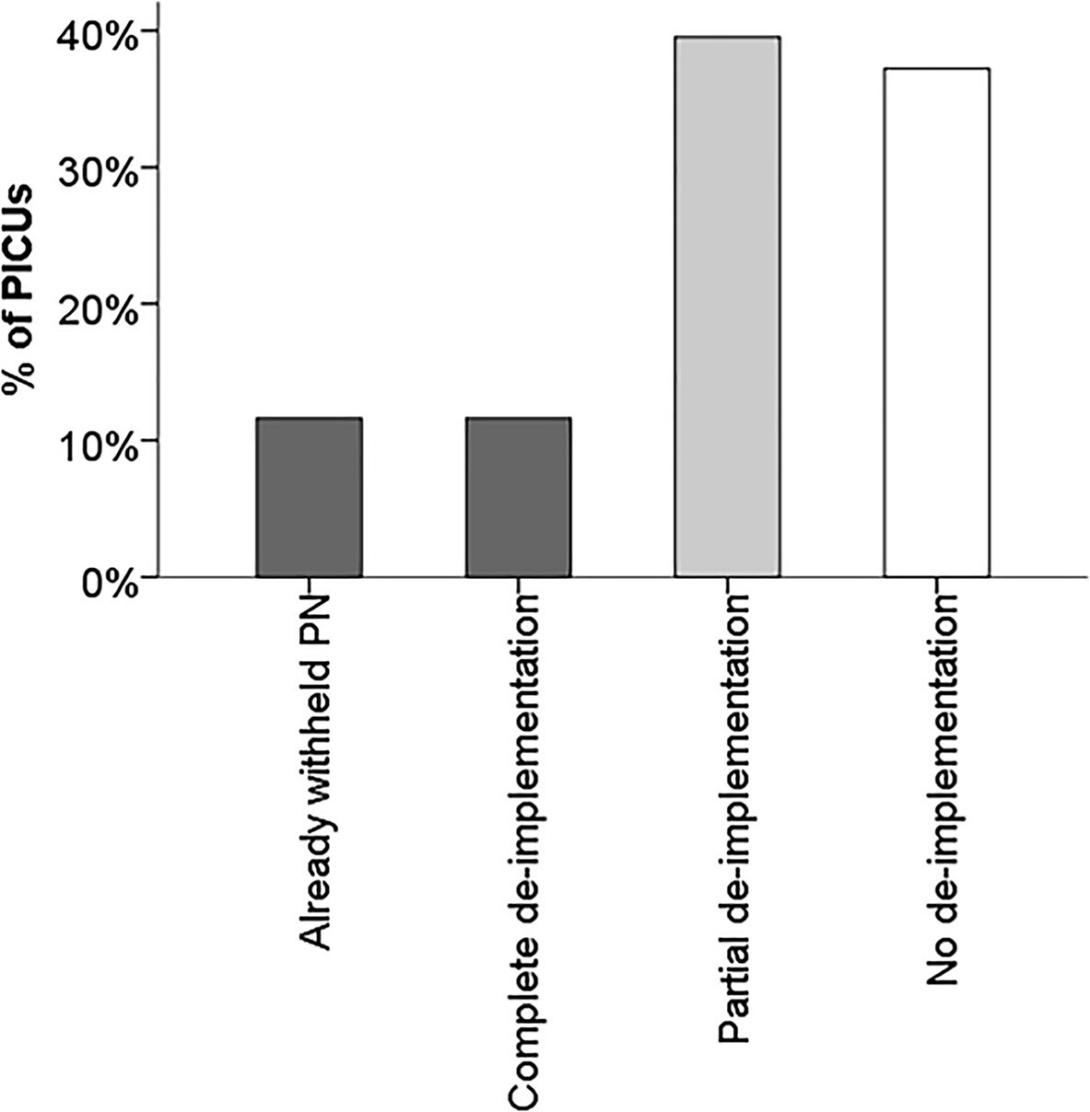
De-implementation of early parenteral nutrition during the first week of paediatric critical illness. PICU = paediatric intensive care unit; PN = parenteral nutrition

Of the 43 respondents who have read the article, 9 (21%) interpreted the level of evidence of the PEPaNIC trial as level 1, 25 (58%) as level 2, and 9 (21%) as level 3. Furthermore, 8 (19%) of these 43 respondents interpreted the grade of recommendation as A (shall be recommended), 17 (39%) as B (should be recommended), and 18 (42%) as 0 (can/may be recommended). These 43 respondents all completed the final part of the survey questions on de-implementation of early PN initiation in their PICU (Fig. 1). Complete de-implementation of early PN initiation, due to the results of PEPaNIC, was reported by 12% (5 of 43) and another 5 (12%) declared to already withhold PN during the first week prior to PEPaNIC (Fig. 4). Partial de-implementation was asserted by 17 (40%) of the respondents (Fig. 4). Of these 17 respondents, 16 reported to give PN during the first week only in specific patient groups (11 to neonates, 11 to undernourished children, and 4 to other, unspecified patients), and 3 respondents declared to have postponed the timing of initiation and/or decreasing the amount of amino acids or lipids. Sixteen (37%) of the 43 PICUs reported no de-implementation, and continued to administer PN early during PICU admission. Ten of these PICUs would start PN within 48 h after admission, of which 6 within 24 h.

### Associations between PICU/respondent characteristics and de-implementation

The degree of de-implementation within the characteristics of the PICUs/respondents is described in Table 2. Higher proportions of complete de-implementation were observed in PICUs from which the respondent rated the level of evidence and grade of recommendation high as compared with those PICUs who rated them lower (Additional file 3: Table S2).

**Table 2:** Barriers for de-implementation (> 1 answer per PICU possible) in the 33 PICUs that have partially or not de-implemented early administration of PN

Barriers	No of PICUs (*n* = 33)
Safety issues	
Not convinced of the safety and/or efficacy in undernourished children	17
Convinced that critically ill children need amino acids in the acute phase of illness	15
Not convinced of the safety and/or efficacy in neonates	11
Convinced that critically ill children need lipids in the acute phase of illness	6
Not convinced of the safety in general	4
Convinced that critically ill children need more glucose in the acute phase of illness	2
Confirmation of results	
Waiting for updated international guidelines^a^	11
Waiting for replicating studies	11
Waiting for long term results	8
Don’t consider these results to be cost-effective	1
Structural reasons	
Non-consensus within staff	9
Other^b^	5
Lack of nutritional protocol	2
Because of logistic reasons (i.e. arrangements with pharmacy)	1
Total number of reasons	103

*PICU* paediatric intensive care unit, *PN* parenteral nutrition

^a^Respondents from Europe: *n* = 7, North America: *n* = 2, South America: *n* = 2 and Africa: *n* = 1

^b^Provided answers: the PEPaNIC results are not generalizable to our PICU: *n* = 3; PN is administrated rarely in our centre: *n* = 1; we are currently changing our PN strategies: *n* = 1

### Barriers for de-implementation

As familiarity of study results are a condition of studying de-implementation, we started off with making this distinction. Of the respondents, only 65% was familiar with the PEPaNIC study and only 43 (53%) had actually read the article. Of these 43, 33 respondents reported no or partial de-implementation and were asked for reasons not to adopt withholding PN during the first week (Fig. 1). The most distinct arguments were those that addressed the safety of postponing PN. The perception that withholding PN would be harmful to children who were undernourished on admission (barrier for 17 respondents, 52%) and neonates (barrier for 11 respondents, 33%) were important barriers. Another major concern was the conviction that parenteral amino acids should be provided during the acute phase of critical illness (mentioned by 15 respondents, 46%). Further arguments represented the need for additional confirmation of the results from the PEPaNIC trial: waiting for replicating studies (11 respondents; 33%), waiting for updated international guidelines (11 respondents; 33%), and waiting for long term outcome results (8 respondents; 24%) (Table 2). Interestingly, 9 (27%) respondents reported that the results from the PEPaNIC trial were discussed within their staff but this had not led to de-implementation of early PN initiation because of lack of consensus (Table 2).

## Discussion

This survey showed that nutritional practices continue to vary greatly among PICUs as was previously reported [3]. Despite the dearth of evidence in the field of nutritional support in the PICU, in the current survey only about two-thirds of the respondents asserted to be familiar with the results from the PEPaNIC trial and approximately half had read the article. Among these respondents, PN was completely withheld during the first week in almost a quarter of the PICUs, and most PICUs had partially de-implemented early PN initiation, which meant predominantly that early PN would only be given to specific patient groups. Reported barriers for de-implementation were predominantly based on the conviction that PN during the first week of critical illness is necessary in neonates and undernourished children, and especially amino acids were viewed to be essential.

Although this de-implementation rate might be considered low, it is to be expected given the relative short time between publication of PEPaNIC and the survey (approximately 1 year). It has been shown that it takes more than a decade from publication to implementation into practice [13, 14]. An important first step in this process is to create awareness of new insights [15]. Interestingly, our survey pointed out that even if the existing evidence in the field is scarce and new results from a large, international study are published in a high-impact, open access journal, only two-thirds of the PICUs was aware of these results.

Besides awareness of new results, (de-)implementation depends on inhibiting and supporting factors. Previous studies have identified the following influences: believe in the benefits for the targeted population, financial implications, organizational structure, caregiver’s motivation to change current practice, feasibility, quality of the evidence, credibility of the working group, relevance and generalizability of the research [16–19]. Indeed, most of these factors were mentioned in our survey as arguments not to change current practice. We will discuss those barriers/facilitators that could guide us to enhance early de-implementation.

In our survey, 76% still administered PN during the first week to all critically ill children or specific patient groups, because they believed in the benefit of early initiation of PN. Despite the fact that early-PN appeared to be even more harmful in neonates than in older children, and more harmful in children at the highest risk of malnutrition, as was already reported in the PEPaNIC article [6], neonates and undernourished children were predominant barriers. After the survey, additional detailed subgroup analyses of neonates and undernourished children were published, which showed that withholding PN was clinically beneficial in these patients as well [20, 21]. Concerns on withholding PN in critically ill children might be explained by several assumptions. Since undernourishment on admission has been associated with worse clinical outcomes, it is assumed that providing (parenteral) nutrition can improve clinical outcome by promoting anabolism. In small RCTs, higher provision of energy and protein/amino acids resulted in a positive protein balance [22, 23]. Subsequently, it was assumed that this would also lead to improved clinical outcome. These assumptions regarding PN might have reduced the faith in the controversial results from the PEPaNIC study, which is also reflected in a number of respondents who requested for repeat studies. Currently, we could identify one single centre RCT on ClinicalTrials.gov, which is designed to randomize 80 critically ill children to receive supplemental PN within 12 or 96 h after admission [24]. However, for clinicians working in combined adult/paediatric ICUs, PEPaNIC could have been considered as a repeat study. Withholding PN for a week in critically ill adults has been included in the ‘choosing wisely campaign’, a list made by specialty societies of possible unnecessary healthcare recommendations [25]. This might explain why PN was completely withheld in critically ill children during the first week in all of the combined adult/paediatric ICUs. Additionally, since evidence for withholding PN during the first week in critically ill adults has already been published first in 2011 [26], the time between evidence from research and de-implementation in practice might play a role. Furthermore, a significant proportion of the respondents mentioned the request for updated guidelines. When the survey was distributed, the most recent international guidelines were developed in 2005 and 2009. In the meantime, these guidelines have been updated by the leading expert nutrition societies [27, 28], which means that the time between previous and current versions of the guidelines was 8 to 13 years. The fact that updated guidelines were awaited by a significant proportion of respondents stresses the importance of up-to-date guidelines. Hence, more frequent updates of the international guidelines might enhance (de-)implementation.

Despite the factors that hamper de-implementation, we have observed a shift in the timing of initiation of PN in critically ill children. In 2013, a worldwide survey was conducted, addressing nutritional practices in the PICU [3]. In this survey, the majority (55%) of the PICUs reported to start PN within 48 h, and 20% within 24 h. Furthermore, PN was completely withheld in only 3.5% of the PICUs before the PEPaNIC results were published [3]. Comparing these results to the results of our study, there seems to be a shift towards initiation of PN between day 2 to 7 and an increase in complete de-implementation of early PN, although this cannot be concluded confidently as the responding PICUs were not exactly the same.

Limiting the delay in de-implementation is of particular importance in case of harm by an intervention ─ which was the case in early-PN ─ or cost-ineffectiveness. Based on our results and existing literature, de-implementation might be accelerated by increasing awareness, gaining trust on the efficacy and safety of stopping the intervention, and facilitating up-to-date international guidelines. An important aspect to take into account is that the personal willingness and readiness to change a practice differs widely, which is illustrated by the ‘theory of the diffusion of innovation’ by Rogers et al. [15] According to this theory, the PICUs who had de-implemented early PN in our survey could be the ‘Early Adopters’, who generally have the highest degree of opinion leadership [15]. Hence, the next step to increase awareness and gain support, demands the Innovators and Early Adopters to distribute the knowledge within their networks. Furthermore, the concerns on the efficacy and safety of stopping the intervention (in our case withholding PN) should be addressed if possible. Since the launch of this survey, several secondary analyses have investigated the main concerns, such as the harm associated with administration of amino acids [29], the efficacy and safety of withholding PN in undernourished children [21] and neonates [20], the long-term effects on physical and neuropsychological functions [30], and the cost-effectiveness of withholding PN [31]. All these new findings were supportive for de-implementation of early-PN. Additionally, underlying mechanisms are currently explored [32]. Finally, since many clinical practices depend on the international opinion, de-implementation might be accelerated if the international guidelines would be revised more frequently in order to cover the most up-to-date evidence.

The strength of our study is the widespread responses from 39 countries. However, some limitations should also be addressed. First, responses from 81 PICUs are a small fraction of all PICUs worldwide, which might decrease generalisability. Possibly, only physicians interested in nutrition might have responded to our survey, which poses a risk of selection bias. Second, some answers from the respondents could potentially have been socially desirable, as this survey has been conducted by the PEPaNIC study group. Furthermore, some respondents gave inconsistent answers. We have analysed all answers as provided by the respondent to avoid incorrect interpretation. And third, with this survey, we have measured theoretical de-implementation based upon the answers of the respondents, without measuring real PN practices. A previous survey addressing nutritional practices in PICUs, in which the questionnaire was followed by a point-prevalence, illustrated that the respondents often overestimated their practices [3].

## Conclusions

One year after the publication of the PEPaNIC trial, only two-thirds of the respondents was aware of the study results. Within this group, complete de-implementation of starting PN in the first week of critical illness was done in 12% of the PICUs worldwide, and partial de-implementation was done in 40% of the PICUs. Another 12% of PICUs already withheld PN during the first week. Important barriers for not de-implementing early PN were concerns on the efficacy and safety of withholding PN, and waiting for updated international guidelines. Increasing the awareness, addressing the intervention-specific questions and more frequently revising the international guidelines might help to accelerate de-implementation of ineffective, unproven or harmful healthcare.

## Additional files

Additional file 1: **Methods 1.** Questionnaire. Questionnaires used for this survey. (DOCX 22 kb)

Additional file 2: **Table S1.** Characteristics and nutritional practices of PICUs of which the respondent had read the article versus those who have not read the article. (DOCX 18 kb)

Additional file 3: **Table S2.** Distribution of the degree of de-implementation within the characteristics of the 43 PICUs/respondents who have answered part D of the questionnaire. (DOCX 18 kb)

## Data Availability

The datasets used and/or analysed during the current study are available from the corresponding author on reasonable request.

## Abbreviations

EN: Enteral nutrition
ESPNIC: European Society of Paediatric and Neonatal Intensive Care
ICU: Intensive Care Unit
NICU: Neonatal Intensive Care Unit
PEPaNIC: Paediatric Early versus Late Parenteral Nutrition in Critically Illness
PICU: Paediatric Intensive Care Unit
PN: Parenteral nutrition
RCT: Randomized controlled trial
SIGN: Scottish Intercollegiate Guidelines Network
WFPICCS: World Federation of Pediatric Intensive and Critical Care Societies
