# Supplementary material for: International survey of De-implementation of initiating parenteral nutrition early in Paediatric intensive care units
Source: BMC Health Serv Res. 2019 Jun 13;19:379. doi: 10.1186/s12913-019-4223-x (PMC6567488; doi:10.1186/s12913-019-4223-x)
Supplement: Supplementary file 1 — Methods 1. Questionnaire. Questionnaires used for this survey. (DOCX 22 kb) [file 12913_2019_4223_MOESM1_ESM.docx]

**International survey of de-implementation of initiating parenteral nutrition early in pediatric intensive care units**

Esther van Puffelen, An Jacobs, Charlotte J.M. Verdoorn, Koen F.M. Joosten, Greet van den Berghe, Erwin Ista, Sascha C.A.T. Verbruggen

**Additional File 1**

Methods 1: Questionnaire

**Methods 1: Questionnaire**

**Part A: General information**

1. What is your country of work?
2. What is the name of your institution?
3. What type of hospital do you work in?

general

university

university-children’s

other, specify

1. What is your profession?

(pediatric) intensivist

anesthesiologist

pediatrician

surgeon

dietician/nutritionist

Nurse/nurse practitioner

other, specify

1. How many years of experience do you have working in a PICU?

1-5

6-10

11-20

>20

1. What type of a PICU do you work in?

multidisciplinary/mixed

surgical

cardiac

medical

other, specify

1. Is the PICU combined with an adult ICU or a neonatal ICU?

Not combined

neonatal

adult

Both neonatal and adult

1. What is the number of pediatric ICU beds in your unit (until age 18 years)?

1-10

11-20

21-30

>30

1. What is the average number of pediatric admissions per year in your unit (until age 18 years)?

1-250

251-500

501-750

751-1000

1001-1250

>1250

1. What is the average proportion of mechanically (invasive) ventilated pediatric patients per year in your unit (until age 18 years)?

<25%

25-50%

>50-75%

>75%

**Part B: Parenteral nutrition in your PICU**

*Please fill in your current practice regarding PN for critically ill pediatric patients.*

1. Is there a nutritional protocol regarding PN used in your PICU?

Yes / No

If Yes 🡪 after C, answer D1

If No 🡪 after C, answer D2

1. What is the basis of your nutritional protocol?

International guideline 🡪 go to B3

National guideline 🡪 go to B4

Opinion of the staff 🡪 go to B4

1. Which guideline?

European Society for Clinical Nutrition and Metabolism (ESPEN) and the European Society for Paediatric Gastroenterology Hepatology and Nutrition (ESPGHAN)

American Society for Parenteral and Enteral Nutrition (A.S.P.E.N.)

Adult international guideline

1. How much glucose is administered during the first 12-24 hours of admission:

- <10 kg 1-4 mg/kg/min (1.4-5.8 g/kg/day) 4-6 mg/kg/min (5.8-8.6 g/kg/day) 6-8 mg/kg/min (8.6-11.5 g/kg/day) 8-10 mg/kg/min (11.5-14.4 g/kg/day)

- 10-30 kg 1-4 mg/kg/min (1.4-5.8 g/kg/day) 4-6 mg/kg/min (5.8-8.6 g/kg/day) 6-8 mg/kg/min (8.6-11.5 g/kg/day) 8-10 mg/kg/min (11.5-14.4 g/kg/day)

- >30 kg 1-4 mg/kg/min (1.4-5.8 g/kg/day) 4-6 mg/kg/min (5.8-8.6 g/kg/day) 6-8 mg/kg/min (8.6-11.5 g/kg/day) 8-10 mg/kg/min (11.5-14.4 g/kg/day)

1. At what point would you start amino acids in a child (expected to be) intolerable to enteral feeds?

< 24 hours

< 48 hours

2-4 days

4-7 days

>7 days

1. At what point would you start lipids in a child (expected to be) intolerable to enteral feeds?

< 24 hours

< 48 hours

2-4 days

4-7 days

>7 days

1. When enteral nutrition is provided but is insufficient to meet target goals, would PN be added?

No

Yes, always

Yes, if enteral nutrition covers < 80% of target calories

Yes, if enteral nutrition covers < 50% of target calories

other

1. If PN is given in combination with enteral nutrition: at what moment (percentage of nutritional target achieved by enteral nutrition) is PN stopped?

If enteral nutrition covers 100% of target calories

If enteral nutrition covers > 80% of target calories

If enteral nutrition covers > 50% of target calories

Other

1. How is PN provided in your institution?

Pharmacy-customized, age/weight specific

Commercial mixed bags

Other

1. Is it possible to administer PN without a central venous line? Yes / No
2. Do you routinely administer vitamins and trace elements? Yes / No

**Part C: Awareness of the Early versus Late PN in Critically Ill Children (PEPaNIC) study**

*The results of the international, multicenter, randomized, controlled trial Pediatric Early versus Late Parenteral Nutrition in Critical Illness (PEPaNIC) have been published in the New England Journal of Medicine in March 2016. The article and supplemental material have been attached to the invitation email.*

1. Are you familiar with the results of this study? Yes / No

2. Did you read this article before you filled out this survey? Yes / No

3. Did you read the supplemental material before you filled out this survey? Yes / No

4. How would you rate the level of evidence?

1 (high to excellent)

2 (moderate to high)

3 (low)

4 (expert opinion)

5. How would you rate the grade of recommendation?

A (shall be recommended)

B (should be recommended)

0 (can/may be recommended)

GPP (Good Practice Points)

**Part D1: Change in Nutritional Practice**

*In the PEPaNIC study, in children who were allocated to the Late PN group, during the first week of critical illness, PN was withheld completely (meaning: low amounts of glucose (<2 mg/kg/min), no amino acids and no lipids were administered). Late PN resulted in lower percentage of new infections and shorter duration of PICU stay compared to Early PN.*

1. Has the local nutritional protocol of your PICU, concerning the initiation or amount of PN, been changed due to the results of this study?

**No** change. We already withheld PN during the first week 🡪 go to D9, after D: go to E4

**No** change. We still administer PN during the first week in all children 🡪 go to D9

**Yes**, we have changed our practice. We now withhold PN during the first week 🡪 go D6 & D8, after D: go to E4

**Yes**, we have changed our protocol partially or only in specific patients. We withhold or decreased component(s) of PN 🡪 go to D2

2. Please specify: during the first week of critical illness in children… (multiple choice)

We still administer PN in the first week only in specific patient groups (i.e. neonates, malnourished children, specific diseases) 🡪 go to D3

We withhold or decrease only some of the macronutrients (glucose, amino acids, lipids) 🡪 go to D5

Other, namely …. 🡪 go to D5

3. Which patient group(s) continued receiving PN during the first week? (multiple choice)

neonates (<1 month)

malnourished children

other, namely …

4. At which day was/is PN started in this patient group?

Neonates:

< 24 hours

< 48 hours

2-4 days

4-7 days

>7 days

Malnourished children:

< 24 hours

< 48 hours

2-4 days

4-7 days

>7 days

Other:

< 24 hours

< 48 hours

2-4 days

4-7 days

>7 days

🡪 go to D9

5. Did you change the administration of parenteral amino acids during the first week due to the results of this study? (multiple choice)

No 🡪 go to D7

Yes, we have changed the timing of initiation of amino acids 🡪 go to D6

Yes, we have changed the amount of amino acids 🡪 go to D7

6. At which day were amino acids started before the change in protocol:

< 24 hours

< 48 hours

2-4 days

4-7 days

>7 days

7. Did you change the administration of parenteral lipids during the first week due to the results of this study? (multiple choice)

No 🡪 go to D9

Yes, we have changed the timing of initiation of lipids 🡪 go to D8

Yes, we have changed the amount of lipids 🡪 go to D9

8. At which day were parenteral lipids started before the change in protocol:

< 24 hours

< 48 hours

2-4 days

4-7 days

>7 days

9. Did you lower the amount of glucose administered intravenously during the first week due to the results of this study? Yes / No

10. Keeping the results of the study in mind, when a child deteriorates clinically after the first week (i.e. sepsis), would you then discontinue PN? Yes / No.

**Part D2: Change in Nutritional Practice**

*In the PEPaNIC study, in children who were allocated to the Late PN group, during the first week of critical illness, PN was withheld completely (meaning: low amounts of glucose (<2 mg/kg/min), no amino acids and no lipids were administered). Late PN resulted in lower percentage of new infections and shorter duration of PICU stay compared to Early PN.*

1. Has the local nutritional practice of your PICU, concerning the initiation or amount of PN, been changed due to the results of this study?

**No** change. We already withheld PN during the first week 🡪 go to D9, after D: go to E4

**No** change. We still administer PN during the first week in all children 🡪 go to D9

**Yes**, we have changed our practice. We now withhold PN during the first week 🡪 go D6 & D8, after D: go to E4

**Yes**, we have changed our practice partially or only in specific patients. We withhold or decreased component(s) of PN 🡪 go to D2

2. Please specify: during the first week of critical illness in children… (multiple choice)

We still administer PN in the first week only in specific patient groups (i.e. neonates, malnourished children, specific diseases) 🡪 go to D3

We withhold or decrease only some of the macronutrients (glucose, amino acids, lipids) 🡪 go to D5

Other, namely …. 🡪 go to D5

3. Which patient group(s) continued receiving PN during the first week? (multiple choice)

neonates (<1 month)

malnourished children

other, namely …

4. At which day was/is PN started in this patient group?

Neonates:

< 24 hours

< 48 hours

2-4 days

4-7 days

>7 days

Malnourished children:

< 24 hours

< 48 hours

2-4 days

4-7 days

>7 days

Other:

< 24 hours

< 48 hours

2-4 days

4-7 days

>7 days

🡪 go to D9

5. Did you change the administration of parenteral amino acids during the first week due to the results of this study? (multiple choice)

No 🡪 go to D7

Yes, we have changed the timing of initiation of amino acids 🡪 go to D6

Yes, we have changed the amount of amino acids 🡪 go to D7

6. At which day were amino acids started before the change in practice:

< 24 hours

< 48 hours

2-4 days

4-7 days

>7 days

7. Did you change the administration of parenteral lipids during the first week due to the results of this study? (multiple choice)

No 🡪 go to D9

Yes, we have changed the timing of initiation of lipids 🡪 go to D8

Yes, we have changed the amount of lipids 🡪 go to D9

8. At which day were parenteral lipids started before the change in practice:

< 24 hours

< 48 hours

2-4 days

4-7 days

>7 days

9. Did you lower the amount of glucose administered intravenously during the first week due to the results of this study? Yes / No

10. Keeping the results of the study in mind, when a child deteriorates clinically after the first week (i.e. sepsis), would you then discontinue PN? Yes / No.

**Part E: Reasons for not implementing Late PN**

1. What is/are reason(s) for not withholding PN in your PICU during the first week of critical illness in children? (multiple choice)

waiting for replicating studies

not convinced of the safety

not convinced of the safety and/or efficacy in neonates

not convinced of the safety and/or efficacy in malnourished children

convinced that critically ill children need amino acids in the acute phase of illness

convinced that critically ill children need more glucose in the acute phase of illness

convinced that critically ill children need lipids in the acute phase of illness

waiting for long term results.

don’t consider these results to be cost-effective

waiting for updated international guidelines

lack of nutritional protocol

non-consensus within staff

Because of logistic reasons (i.e. arrangements with pharmacy) (🡪 go to E2)

Other, namely …..

All answers (except logistic reasons)🡪go to E4

2. When enteral nutrition is insufficient to meet nutritional targets, do you intend to withhold PN during the first week of critical illness in the future?

No, I intend to start PN as soon as possible

Yes, I intend to withhold PN for less than 7 days in the future (🡪 go to E3)

Yes, I intend to withhold PN during the first week in the future (🡪 go to E4)

3. At which day do you intend to start PN in the future?

< 24 hours

< 48 hours

2-4 days

4-7 days

>7 days

4. Do you have any comments on this survey? (not mandatory)

5. If you would like to be informed about the results of this survey, please fill in your name and email-address. (not mandatory)
