# Supplementary material for: International survey of De-implementation of initiating parenteral nutrition early in Paediatric intensive care units
Source: BMC Health Serv Res. 2019 Jun 13;19:379. doi: 10.1186/s12913-019-4223-x (PMC6567488; doi:10.1186/s12913-019-4223-x)
Supplement: Supplementary file 2 — Table S1. Characteristics and nutritional practices of PICUs of which the respondent had read the article versus those who have not read the article. (DOCX 18 kb) [file 12913_2019_4223_MOESM2_ESM.docx]

**International survey of de-implementation of initiating parenteral nutrition early in pediatric intensive care units**

Esther van Puffelen, An Jacobs, Charlotte J.M. Verdoorn, Koen F.M. Joosten, Greet van den Berghe, Erwin Ista, Sascha C.A.T. Verbruggen

**Additional File 2**

**Table S1:** Characteristics and nutritional practices of PICUs of which the respondent had read the article versus those who have not read the article

**Table S1: Characteristics and nutritional practices of PICUs of which the respondent had read the article versus those who have not read the article**

| **Characteristic /**  **nutritional practice** | **Have read the article (n=43)** | **Did not read the article (n=38)** |
| --- | --- | --- |
| Continent |  |  |
| Europe | 22 (51.2%) | 17 (44.7%) |
| South America | 8 (18.6%) | 6 (15.8%) |
| Asia | 2 (4.7%) | 10 (26.3%) |
| North America | 8 (18.6%) | 4 (10.5%) |
| Africa | 2 (4.7%) | 0 (0%) |
| Oceania | 1 (2.3%) | 1 (2.6%) |
| Hospital type |  |  |
| University children’s hospital | 26 (60.5%) | 11 (28.9%) |
| University hospital | 10 (23.3%) | 14 (36.8%) |
| General hospital | 6 (14.0%) | 12 (31.6%) |
| Other | 1 (2.3%) | 1 (2.6%) |
| Type of PICU |  |  |
| Multidisciplinary/mixed | 43 (100%) | 32 (84.2%) |
| Medical | 0 (0%) | 4 (10.5%) |
| Cardiac | 0 (0%) | 1 (2.6%) |
| Surgical | 0 (0%) | 1 (2.6%) |
| Combination of PICU |  |  |
| Not combined | 35 (81.4%) | 31 (81.6%) |
| With neonatal ICU | 5 (11.6%) | 5 (13.2%) |
| With adult ICU | 3 (7.0%) | 1 (2.6%) |
| With adult and neonatal ICU | 0 (0%) | 1 (2.6%) |
| Size of PICU |  |  |
| 1-10 beds | 12 (27.9%) | 21 (55.3%) |
| 11-20 beds | 16 (37.2%) | 12 (31.6%) |
| 21-30 beds | 12 (27.9%) | 4 (10.5%) |
| >30 beds | 3 (7.0%) | 1 (2.6%) |
| Paediatric admissions (patients/year) |  |  |
| 1-250 | 3 (7.0%) | 4 (10.5%) |
| 251-500 | 13 (30.2%) | 16 (42.1%) |
| 501-750 | 9 (20.9%) | 9 (23.7%) |
| 751-1000 | 4 (9.3%) | 3 (7.9%) |
| 1001-1250 | 5 (11.6%) | 2 (5.3%) |
| >1250 | 9 (20.9%) | 4 (10.5%) |
| Mechanically ventilated paediatric patients |  |  |
| <25% | 6 (14.0%) | 3 (7.9%) |
| 25-50% | 13 (30.2%) | 18 (47.4%) |
| 50-75% | 14 (32.6%) | 11 (28.9%) |
| >75% | 10 (23.3%) | 6 (15.8%) |
| Add PN if EN is insufficient |  |  |
| No | 2 (4.7%) | 2 (5.3%) |
| Yes, if EN <50% | 13 (30.2%) | 7 (18.4%) |
| Yes, if EN <80% | 21 (4.7%) | 22 (57.9%) |
| Yes, always | 5 (11.6%) | 5 (13.2%) |
| Other | 2 (4.7%) | 2 (5.3%) |
| Start amino acids |  |  |
| <24 hours | 12 (27.9%) | 9 (23.7) |
| <48 hours | 5 (11.6%) | 11 (28.9%) |
| 2-4 days | 12 (27.9%) | 9 (23.7%) |
| 4-7 days | 10 (23.3%) | 7 (18.4%) |
| >7 days | 4 (9.3%) | 0 (0.0%) |
| Start lipids |  |  |
| <24 hours | 9 (20.9%) | 5 (13.2%) |
| <48 hours | 9 (20.9%) | 11 (28.9%) |
| 2-4 days | 11 (25.6%) | 10 (26.3%) |
| 4-7 days | 11 (25.6%) | 11 (28.9%) |
| >7 days | 3 (7.0%) | 1 (2.6%) |

PICU = paediatric intensive care unit; ICU = intensive care unit; PN = parenteral nutrition
