# Supplementary material for: International survey of De-implementation of initiating parenteral nutrition early in Paediatric intensive care units
Source: BMC Health Serv Res. 2019 Jun 13;19:379. doi: 10.1186/s12913-019-4223-x (PMC6567488; doi:10.1186/s12913-019-4223-x)
Supplement: Supplementary file 3 — Table S2. Distribution of the degree of de-implementation within the characteristics of the 43 PICUs/respondents who have answered part D of the questionnaire. (DOCX 18 kb) [file 12913_2019_4223_MOESM3_ESM.docx]

**International survey of de-implementation of initiating parenteral nutrition early in pediatric intensive care units**

Esther van Puffelen, An Jacobs, Charlotte J.M. Verdoorn, Koen F.M. Joosten, Greet van den Berghe, Erwin Ista, Sascha C.A.T. Verbruggen

**Additional File 3**

**Table S2:** Distribution of the degree of de-implementation within the characteristics of the 43 PICUs/respondents who have answered part D of the questionnaire

**Table S2: Distribution of the degree of de-implementation within the characteristics of the 43 PICUs/respondents who have answered part D of the questionnaire**

| **Characteristic** | **No de-implementation** | **Partial de-implementation** | **Complete de-implementation** | **Already withheld PN** |
| --- | --- | --- | --- | --- |
| Continent |  |  |  |  |
| Europe | 2 (9%) | 8 (36%) | 8 (36%) | 2 (9%) |
| South America | 2 (25%) | 4 (50%) | 0 (0%) | 2 (25%) |
| Asia | 2 (100%) | 0 0%) | 0 (0%) | 0 (0%) |
| North America | 3 (39%) | 3 (38%) | 1 (13%) | 1 (13%) |
| Africa | 1 (50%) | 1 (50%) | 0 (0%) | 0 (0%) |
| Oceania | 0 (0%) | 1 (100%) | 0 (0%) | 0 (0%) |
| Combination of PICU |  |  |  |  |
| Not combined | 14 (40%) | 15 (43%) | 3 (9%) | 3 (9%) |
| With neonatal ICU | 2 (40%) | 2 (40%) | 0 (0%) | 1 (20%) |
| With adult ICU | 0 (0%) | 0 (0%) | 2 (67%) | 1 (33%) |
| With adult and neonatal ICU | 0 (0%) | 0 (0%) | 0 (0%) | 0 (0%) |
| Size of PICU |  |  |  |  |
| 1-10 beds | 5 (42%) | 4 (33%) | 1 (8%) | 2 (7%) |
| 11-20 beds | 5 (31%) | 6 (38%) | 3 (19%) | 2 (13%) |
| 21-30 beds | 6 (50%) | 5 (42%) | 1 (8%) | 0 (0%) |
| >30 beds | 0 (0%) | 2 (67%) | 0 (0%) | 1 (33%) |
| Experience of respondent (years) |  |  |  |  |
| 1-5 | 0 (0%) | 3 (50%) | 2 (33%) | 1 (17%) |
| 6-10 | 5 (71%) | 2 (29%) | 0 (0%) | 0 (0%) |
| 11-20 | 6 (46%) | 3 (23%) | 1 (8%) | 3 (23%) |
| >20 | 5 (29%) | 9 (53%) | 2 (12%) | 1 (6%) |
| Nutritional protocol present |  |  |  |  |
| Yes | 12 (41%) | 10 (35%) | 4 (14%) | 3 (10%) |
| No | 2 (29%) | 7 (50%) | 1 (7%) | 2 (14%) |
| Rated level of evidence |  |  |  |  |
| 4 | 0 (0%) | 0 (0%) | 0 (0%) | 0 (0%) |
| 3 | 7 (78%) | 2 (22%) | 0 (0%) | 0 (0%) |
| 2 | 7 (28%) | 11 (44%) | 2 (8%) | 5 (20%) |
| 1 | 2 (22%) | 4 (44%) | 3 (33%) | 0 (0%) |
| Rated grade of recommendation |  |  |  |  |
| Good Practice Points | 0 (0%) | 0 (0%) | 0 (0%) | 0 (0%) |
| 0 | 10 (56%) | 6 (33%) | 0 (0%) | 2 (11%) |
| B | 3 (18%) | 9 (53%) | 2 (12%) | 3 (18%) |
| A | 3 (38%) | 2 (25%) | 3 (38%) | 0 (0%) |

PICU = paediatric intensive care unit; ICU = intensive care unit; PN = parenteral nutrition
